# Effects of Zn, Mg and Cu Doping on Oxidation Reaction of Al (111) Surface

**DOI:** 10.3389/fchem.2022.930900

**Published:** 2022-06-29

**Authors:** Hua Ji, Keliang Ren, Jia Yang, Yating Zhang, Guan Wang

**Affiliations:** ^1^ School of Physics and Electronic-Electrical Engineering, Ningxia University, Yinchuan, China; ^2^ School of Mechanical Engineering, Ningxia University, Yinchuan, China

**Keywords:** density functional theory, lithium-ion batteries shell, aluminum alloys, surface doping, oxidation mechanism

## Abstract

Aiming at the performance degradation of lithium-ion batteries due to shell corrosion, the doping of alloy elements Zn, Mg and Cu on Al (111) surface and the effect on oxidation reaction of Al (111) surface were studied by the first-principles calculation method. The results show that Zn, Mg and Cu atoms stably combine with Al atoms, and the surface smoothness is slightly different due to their different radii and electronegativity. The dissociative adsorption of O_2_ molecules is related to the surface doping atoms and O_2_ coverage, while the electron tunneling of underlying metal promotes O_2_ adsorption on the surface. As O_2_ coverage increases, the O atoms adsorbed on the hcp site gradually migrate to the subsurface layer. Zn, Mg, Cu and vacancy defect hinder the migration of the surrounding O atoms to subsurface layer, resulting in different structures and thicknesses of the oxide film near the doped atoms. At the same time, Zn, Mg, and Cu atoms differ in their ability to gain or lose electrons compared with Al atoms, resulting in their different positions on the surface. In addition, the surface work function rises with the increase of O_2_ coverage, and Zn and Cu atoms make the work function increase faster. Finally, according to the research results, it can be inferred that Zn and Mg are the unfavorable factors for the oxidation reaction of Al surface.

## 1 Introduction

As a clean and efficient secondary battery, lithium-ion batteries have been widely used in electric vehicles, energy storage systems, and mobile electronic devices. The battery shell is an important part of the battery. It is not only a simple sealing container, but also has an important impact on the storage performance and safety performance of the battery. During the Application of the battery, the battery shell is easily corroded by the external environment and the electrolyte, thus affecting the service life of lithium battery. Generally, aluminum alloys are used as shell materials in lithium-ion batteries because of their high specific strength, fine formability, heat dissipation, and corrosion resistance. At the early stage of local corrosion, a dense oxide film usually exists on the surface of aluminum alloy. This oxide film is an excellent insulator and prevents corrosion of the substrate. Therefore, many researchers have studied the oxidation process on the Al surface. Popova et al. (Popova et al., 2000) observed the formation of an oxide layer with a thickness of about 2–2.5 nm on the Al (111) surface. Oxide layers were also observed on Cu-9@%Al (111) (Yoshitake et al., 2003), TiAl (111) (Maurice et al., 2005) and Ni_3_Al (111) (Bardi et al., 1992; Addepalli et al., 1999; Degen et al., 2005; Qin et al., 2005; Hamm et al., 2006). Cabrera et al. (Cabrera and Mott, 1949) mentioned that the oxidation process of metals begins with the dissociation of oxygen molecules on metal surface. Many experimental results (Brune et al., 1992; Komrowski et al., 2001; Cai et al., 2011) also confirm that O atoms rather than O_2_ molecules are adsorbed on Al surface. Cai et al. (Cai et al., 2011; Cai et al., 2012) studied the oxide film on Al surface at room temperature by X-ray photoelectron spectroscopy (XPS), and found that the oxide film on Al surface becomes thicker with the increase of oxygen partial pressure. While Baran et al. (Baran et al., 2014) proposed the limit thickness of oxide films formed on aluminum, and compared the first-principles results with the traditional Cabrera-Mott (CM) model, which is a classical continuum model. The present results support experimental estimates of the Mott potential and film thicknesses. Pashutski et Al. (Pashutski et al., 1989) studied the adsorption of O_2_ on Al (100) surface at 80 K by XPS and Auger electron spectroscopy (AES). The results showed that Al_x_O_y_ oxide was formed at low coverage (x:y = 3:1–1:1), at high coverage or heated to room temperature, the oxide layer transforms into common Al_2_O_3_. In the presence of water vapor, the Al surface oxides are hydroxylated to form an “Al(OH)_3_-Al_2_O_3_” double-layer structure (Cai et al., 2012).

From the above conclusions, it can be seen that researchers have conducted extensive studies on the mechanism and microscopic process of metal oxidation through experiments and theoretical simulations. The oxidation behavior of high-temperature structural materials is also one of the research contents that researchers are interested in. Pan et al. (Pan and Pu, 2020) study the oxidation mechanism of Mo_5_SiB_2_. Pu et al. (Pu and Pan, 2022) reveal the oxidation behavior of the prefect Mo_5_Si_3_. Meanwhile, Pan et al. (Pan and Wang, 2018; Pan and Wen, 2018; Wang et al., 2018; Pan et al., 2020a; Pan et al., 2020b; Pu and Pan, 2022) also discuss the influence of alloying element doping on the oxidation properties of high-temperature materials. The doped transition metal elements (Pt and Pd) narrow the band gap, which further promotes the electron transport of the catalyst. And the doped elements enhance the catalytic hydrogen evolution performance of the heterojunction (Chen and Pan, 2022). It can be seen that the doping of alloying elements can affect the oxidation reaction and surface properties of materials. The structure determines the property, and the surface structure affects the surface oxidation process. Surface doping, vacancies and substitutions are all typical defect-type of surface structures, which change the surface activity and affect the interaction between adsorbates and surface, and are an important factor in the oxidation process.

As the main alloying elements of aluminum alloys, Zn, Mg and Cu atoms provide precipitation strengthening for alloys, and have an important influence on oxidation of alloys. Under controlled conditions, if doping alloying elements can increase the aluminum oxide thickness on Al surface, the corrosion resistance of lithium-ion battery shell can be effectively improved. Therefore, the doping of alloying elements Zn, Mg and Cu on Al surface and their effects on the oxidation process of Al surface were studied by first principles, in order to find out which doping elements are conducive to the oxidation of Al surface. The results provide help for further understanding of the oxidation and protection mechanism of Al-based products.

## 2 Computational Details

DFT calculations were performed by the Cambridge Serial Total Energy Package (CASTEP). And the first-principles plane wave pseudopotential method based on DFT was adopted (Clark et al., 2005). Exchange-correlation energy was estimated by the Perdew Burke Ernzerhof (PBE) functional of the generalized gradient approximation (GGA) (Perdew et al., 1996). The bulk Al lattice parameter was calculated to be 0.4045 nm that agreed excellently with the experimental value 0.4049 nm (Straumanis and Woodward, 1971). Al (111) is a close-packed plane and stable, it is beneficial to explain the advancement of this topic. Therefore, in this work, the surface adsorption calculation were conducted on six layers slabs of Al (111) with a 1.2 nm vacuum gap. After testing, the atomic layer thickness was sufficient to avoid interactions between the outermost atoms. The Monkhorst-Pack *k*-point of (4 × 4 × 1) and the plane-wave energy cutoff of 520 eV were used to adsorption calculations. In the optimization process, the adsorbates and the three uppermost surface layers were allowed to move freely, while the bottom three layers were fixed. Periodic boundary conditions were set to make it an infinite periodic system. The convergence thresholds for total energy and maximum force are 1.0 × 10^–5^ eV atom^−1^ and 0.03 eV/Å, respectively. Al (s2 p1), Zn (d10 p2), Mg (s2), Cu (d10 p1) are treated as valence electrons.

The surface energy $\gamma_{\mathrm{surf}}$ refers to Gibbs free energy per unit area, which is a parameter to measure the surface stability of materials, and is calculated by the following expression.

$$\gamma_{\mathrm{surf}}=\frac{\left(E_{\mathrm{unrelax}}-\sum_{i}n_{i}\mu_{i}\right)}{2A}+\frac{\left(E_{\mathrm{relax}}-E_{\mathrm{unrelax}}\right)}{A}$$ (1)

Where $E_{\mathrm{unrelax}}$ is the total energy of surface model before optimization; $E_{\mathrm{relax}}$ is the total energy of surface model after optimization; $\sum_{i}n_{i}\mu_{i}$ is the energy of the surface atoms in the bulk phase; $n_{i}$ is the number of atoms in surface model; $\mu_{i}$ is the energy of one atom in a single crystal; A is the surface area. In general, the lower the surface energy, the more stable the structure is.

The doping energy $E_{\mathrm{im}}$ of doping atom is calculated as follows:

$$E_{\mathrm{im}}=E_{\mathrm{total}}-E_{\mathrm{surf}}^{\mathrm{Al}}-E_{m}$$ (2)

Where, $E_{\mathrm{total}}$ is the total energy of doping model; $E_{\mathrm{surf}}^{\mathrm{Al}}$ is the energy of model after removing the doping atoms; $E_{m}$ is the energy of the doping atoms. The doping energy is negative, indicating that the doping atoms can stably combine with Al atoms on the model surface.

The work function Φ describes the energy required for electrons to escape from material surface, and reflects the stability of surface electrons (Da Silva, 2005). The larger the work function, the more stable the surface electrons are. The work function is calculated by the following expression.

$$\Phi =E_{\mathrm{vacuum}}-E_{\mathrm{Fermi}}$$ (3)

Where $E_{\mathrm{vacuum}}$ is the vacuum energy and $E_{\mathrm{Fermi}}$ is the corresponding Fermi energy. $E_{\mathrm{vacuum}}$ was calculated as the averaged electrostatic potential in the middle of the vacuum region, which was possible by setting surface alloy on the both sides of the slab.

The adsorption energy $E_{\mathrm{ad}}$ is calculated by the following expression.

$$E_{\mathrm{ad}}=E_{\mathrm{ads}+\mathrm{surf}}-E_{\mathrm{ads}}-E_{\mathrm{surf}}$$ (4)

In the expression, $E_{\mathrm{ads}+\mathrm{surf}}$ , $E_{\mathrm{ads}}$ , $E_{\mathrm{surf}}$ represent the total energy of structure after adsorption, the total energy of adsorbate and the total energy of adsorption surface, respectively. According to the definition of adsorption energy, with the increase of adsorption energy, the interaction between the adsorbate and the surface gradually grows.

## 3 Results and Discussions

### 3.1 Surface Analysis of Doping Models

**FIGURE 1:**
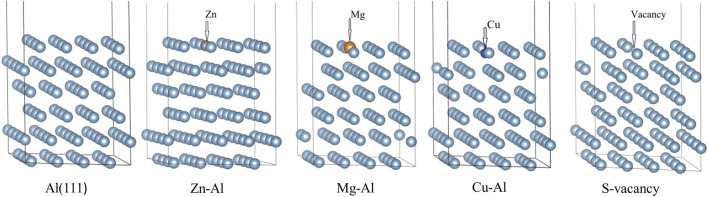
Calculation model of the surface. The structure is composed of a six-layer slab and a 1.2 nm vacuum space, the topmost layer Al atom were substitued by Zn, Mg and Cu atoms. The surface vacancy model is achieved by removing the Al atom.

**FIGURE 2:**
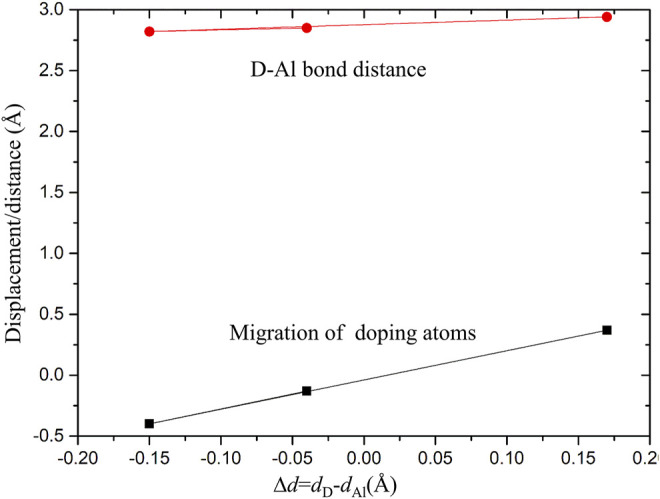
Distortion as a function of ∆*d*. ∆*d* is the difference between the radii of the doping atoms and Al atoms; the red dots are the average distances between doping atoms and surrounding Al atoms; the black squares are the migration displacements of the doping atoms.

After calculation, the work function of cleaning Al (111) surface is 4.0406 eV, which is consistent with experimental result of 4.23 eV (Grepstad et al., 1976) and theoretical calculation result of 4.02 eV (Singh-Miller and Marzari, 2009). It shows that this model can reproduce structural properties of Al (111) surface. In order to understand the effect of alloying elements Zn, Mg, and Cu on adsorption of Al (111) surface, a surface doping model was established by replacing the uppermost Al atom with Zn, Mg, and Cu atoms. It was found by calculation that the surface energy of surface doping model is independent of substitution positions of Zn, Mg and Cu atoms. Meanwhile, in order to avoid the transverse interaction between mirror cells, the replacement positions of Zn, Mg and Cu atoms are shown in Figure 1. The pure Al (111) surface model was defined as Al (111), the Zn-doped model as Zn-Al, the Mg-doped model as Mg-Al, and the Cu-doped model as Cu-Al. In addition, surface vacancy is also a typical defect surface structure. To compare with the surface doping model, a surface vacancy model (S-vacancy) was established. The surface vacancy model was achieved by removing one Al atom in the topmost layer. After geometry optimization, the flatness of model surface is different. As can be seen from Figure 1, Cu atom is slightly embedded in the surface and Mg atom protrudes slightly from the surface. This is due to the difference in the radii and electronegativity of the doping atoms. It can be seen from Figure 2 that with increasing radii difference between the doping atoms and the matrix Al atom, the migration displacement of doping atoms from surface is monotonically increasing. The electronegativity of Cu atom is stronger than that of Al atom, and Cu atom is embedded in the surface. While the electronegativity of Mg atom is weaker than that of Al atom, and Mg atom protrudes from the surface. The radius and electronegativity of Zn atom are similar to those of Al atom, and the model surface is very flat. In addition, after the doping atoms are added, the surrounding Al atoms will also have a displacement. The larger the radii difference between the doping atoms and Al atom is, the larger the distance between the doping atoms and Al atom is, that is, the larger the distortion caused by the doping atoms is. The distortion of Al matrix and different surface flatness will also have an impact on the subsequent oxidation reaction.

Table 1 shows the doping energy, surface energy, work function and charge transfer of each surface doping model and surface vacancy model. According to the calculation results in Table 1, the doping energy of doping atoms in each model is negative, indicating that Zn, Mg and Cu atoms can stably combine with Al atoms on the model surface. And, the surface energy of each model varies with different doping atoms. Compared with pure Al (111) (0.8258J/m^2^), the surface energy of doped Mg and Cu model is lower, while that of surface vacancy model is higher. This indicates that Mg and Cu atoms are more likely to appear on Al (111) surface, while vacancies are less likely to appear on Al (111) surface. In particular, the surface energy of the Zn-doped model is 0.8293 J/m^2^, which is consistent with calculated result of Liu et al. (Liu et al., 2011) (0.844 J/m^2^). In addition, the work functions of all surface doping models and surface vacancy models are larger than that of pure Al (111) surfaces. It is suggested that doping atoms stabilize the surface electrons. The work function of Mg-Al is almost the same as that of Al (111) surface, which should be due to the similar atomic weight and atomic radius of Mg and Al atoms. According to the charge transfer calculated by Bader charge, it is known that surface Al atoms lose electrons to Cu and Zn atoms due to the stronger electronegativity of Cu and Zn compared with Al. And the electronegativity of Mg is weaker than that of Al, so Mg atoms lose electrons to Al atoms. The charge changes of Mg and Cu atoms are the largest, which indicates that interaction between surface Al atoms and Mg and Cu atoms is stronger. This gravitational effect increases the bonding between atoms on the surface. Then, the Mg-Al and Cu-Al surface are also more stable relative to other surfaces. Therefore, the stability order of the surface model is: Mg-Al > Cu-Al > Al (111) > Zn-Al > S-vacancy. The above results show that the stability of the surface depends on the type of doping atoms and their electronic properties.

**TABLE 1:** Doping energy, surface energy, work function and charge transfer of surface doping model and surface vacancy model.

Model	Doping Energy	Surface Energy (J/M^2^)	Work Function (eV)	Charge Transfer
	(eV)			Al→Dopant Atoms
Zn-Al	−0.5515	0.8293	4.0445	0.4014
Cu-Al	−0.6052	0.8133	4.0542	0.8531
Mg-Al	−0.7563	0.8052	4.0407	−1.3402
S-vacancy	—	0.8664	4.0553	—

### 3.2 Adsorption of an O_2_ Molecule on Doping Surface

**FIGURE 3:**
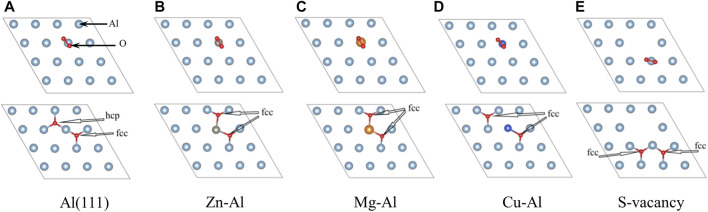
The top view of adsorbed structures of O_2_ on model.

**FIGURE 4:**
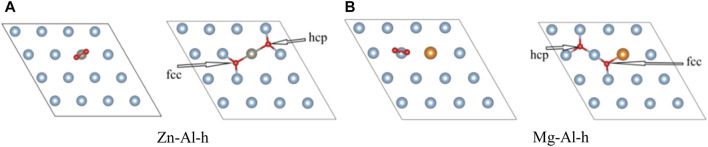
The top view of other adsorption configurations.

In this part, the effect of doping of Zn, Mg and Cu atoms on adsorption of O_2_ molecules on the Al surface is studied. O_2_ molecules are most easily adsorbed on the Al surface when they are parallel to the surface (Zhang et al., 2004). Therefore, O_2_ molecule was placed parallel to the top of doping atoms on model surface (as shown in Figure 3). And O_2_ molecule was relaxed under this constraint. Considering the triplet state of O_2_, all calculations are performed with spin-polarized calculations. After optimization, it is found that O_2_ molecule spontaneously dissociates into O atoms and adsorbs on the model surface, which is consistent with previous research results (Cabrera and Mott, 1949; Brune et al., 1992; Komrowski et al., 2001; Cai et al., 2011). Moreover, most of the two O atoms are adsorbed on the nearest-neighbor fcc sites near Zn, Mg, Cu atoms and surface vacancies, and only one O atom is adsorbed on the hcp site. This suggests that O atoms prefer to adsorb on the fcc sites. In this paper, the adsorption configurations of oxygen in different orientations and adsorption sites were also calculated, and more O atoms were found to be adsorbed on the hcp sites. Two of these examples (Zn-Al-h, Mg-Al-h) are shown in Figure 4, where O atoms are adsorbed on the nearest-neighbor hcp sites. The geometric parameters, work function, charge transfer and adsorption energies of adsorbing one O_2_ molecule in the above 7 configurations are listed in Table 2. It can be clearly seen from Table 2 that the adsorption energy of the fcc site is greater than that of the hcp site. It shows that the adsorption of O atom on the fcc site is more stable than that on the hcp site. This is consistent with the results of Wei et al. (Wei et al., 2016), who believe that the fcc site is the most stable adsorption site for O atoms, and the hcp site is the metastable adsorption site. According to charge transfer from the model surface to O_2_ molecule in Table 2, it can be seen that charge transfer of Zn-Al-h and Mg-Al-h models is larger than that of Zn-Al and Mg-Al models, respectively. It shows that more charges are required for O atom adsorption on the hcp site. In addition, the adsorption energy of Mg-Al, Mg-Al-H and S-Vacancy is greater than that of Al (111), and the adsorption energy of Zn-Al, Zn-Al-h and Cu-Al is smaller than that of Al (111). This indicates that the doping of Mg and the appearance of surface vacancies promotes the absorption of O_2_ molecules on Al (111) surface, while the doping of Zn and Cu delay the adsorption of O_2_ molecules on Al (111) surface. The maximum average distance between Al atom and O atom on the surface is 1.8647 Å, which is in the Al (111) adsorption configuration. And this distance is smaller than the sum of the ionic radii between Al^3+^ (0.535 Å) and O^2-^ (1.4 Å), that is, Al-O is bound in the form of chemical bonds. In the doping model, the chemical bond between Al and O becomes shorter due to the induction effect of doping atoms, which means that the bonding effect between Al and O atoms is enhanced. Meanwhile, comparing the work functions of the models in Table 1 and Table 2, it is found that the work functions of all models increase after adsorption of one O_2_ molecule. This means that the adsorption of O_2_ molecule makes the surface electrons more stable.

**TABLE 2:** Geometrical parameters, work function, charge transfer, and adsorption energies of one O_2_ molecular adsorbed on the surface of model.

Model	Distance (Å) Al-O (average)	Work function (eV)	Charge Transfer	Adsorption energy (eV)
			Al→O_2_	
Al (111)	1.8647	4.0519	3.4922	−8.5247
Zn-Al	1.8124	4.3230	3.1405	−7.9417
Zn-Al-h	1.8070	4.1817	3.1602	−7.5900
Mg-Al	1.8229	4.0606	3.3646	−9.1000
Mg-Al-h	1.8450	4.0639	3.4495	−8.7059
Cu-Al	1.8156	4.1548	3.3043	−8.2892
S-vacancy	1.8630	4.0714	3.4458	−8.9133

**FIGURE 5:**
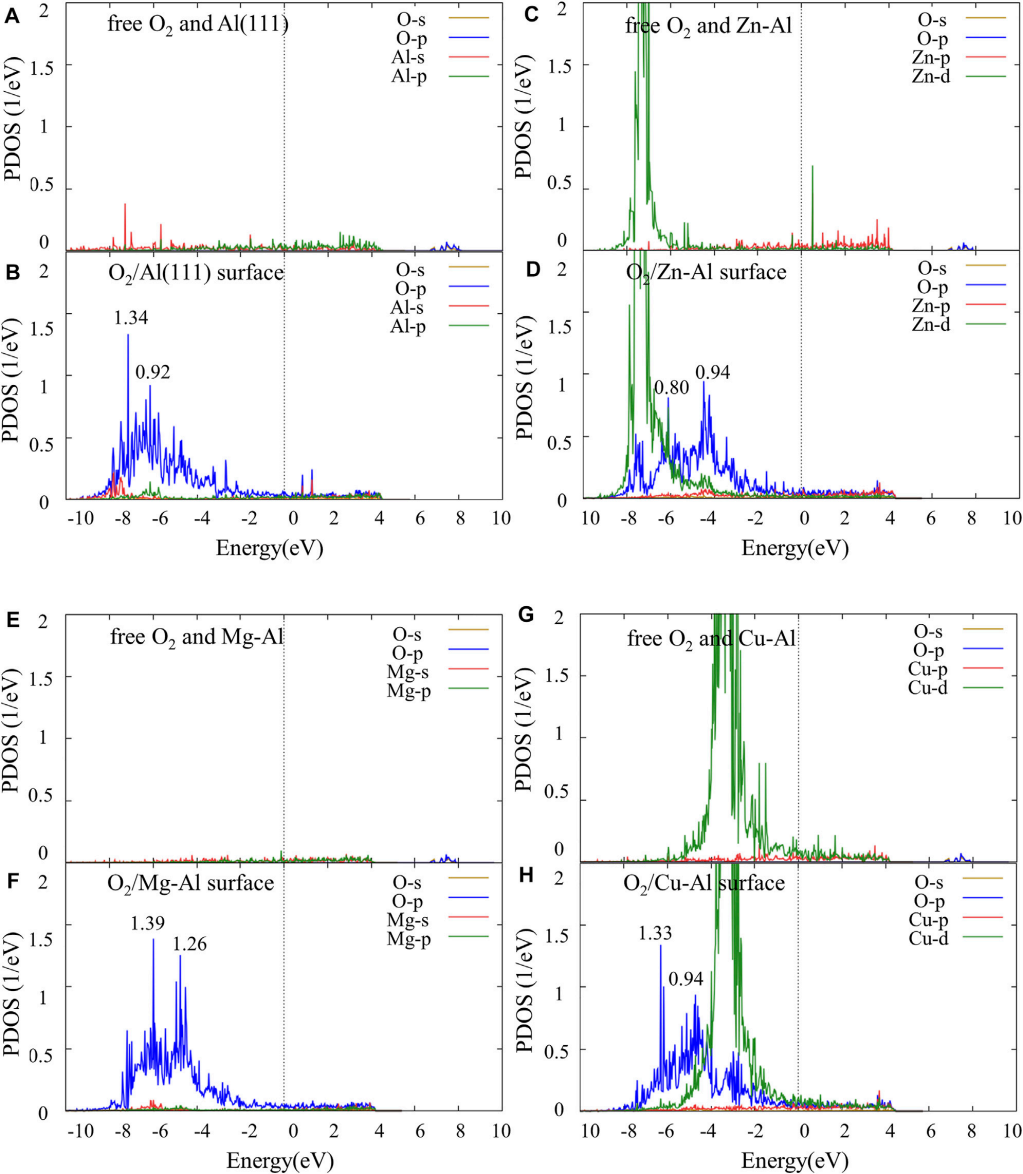
The partial density of states (PDOS) of O_2_ and Al (111) surfaces, Zn-Al surfaces before and after adsorption: **(A)** free O_2_ molecules and Al (111) surface, **(B)** O_2_ molecules adsorbed on Al (111) surface, **(C)** free O_2_ molecules and Zn-Al surface, **(D)** O_2_ molecules adsorbed on Zn-Al surface, **(E)** free O_2_ molecules and Mg-Al surface, **(F)** O_2_ molecules adsorbed on Mg-Al surface, **(G)** free O_2_ molecules and Cu-Al surface, **(H)** O_2_ molecules adsorbed on Cu-Al surface, where the Fermi energy is set to zero.

To further study the interaction mechanism between the top Al atom/doping atom and O atom, the partial density of states (PDOS) of O_2_ molecule and each model surface before and after adsorption was calculated, as shown in Figure 5. As a special case, only the PDOS of the stable adsorption site (fcc site) are given. Comparing with Figures 4A,B, it can be seen that the energy level of O-p orbital moves from high energy region to low energy region, after O atom is adsorbed on Al (111) surface. And O-p orbital has an obvious broadening. Compared with free O_2_ molecules, the adsorbed O_2_ molecules are dissociated into O atoms, which bond with surface Al atoms. Thus, the energy level movement and broadening of the O-p orbital can be attributed to the interaction between O atom and surface Al atoms. In addition, PDOS of the surface Al atoms shows that there is little change in the orbital energy level of Al atoms near the Fermi surface, but there are obvious peak values of Al-s and Al-p orbitals in the low energy region, and orbital hybridization formed with O-p orbital. It is indicated that the adsorption of O_2_ molecule on Al (111) surface is mainly due to the action of O-p orbital and Al-s, Al-p orbital in low energy region (−8.9 eV ∼ −6.2 eV). Similarly, it can be seen from Figure 5 that the energy levels of O-p orbital all moves from high energy region to low energy region after O_2_ molecules are adsorbed on Zn-Al, Mg-Al, and Cu-Al surface. And O-p orbitals have a significantly broadening. Meanwhile, Zn-d, Mg-s and Cu-d orbitals are also hybridized with O-p orbitals. This indicates that Zn-d, Mg-s and Cu-d orbitals interact with O-p orbitals, causing the O atoms to adsorb on the surface. After the adsorption of O_2_ molecules on each model surface, the density peak of O-p orbital states were compared. It can be seen that the O-p orbital peak of density of states on the Mg-Al surface is the largest (1.39). It is indicated that the interaction between O atoms and Mg-Al surface should be the strongest. The O-p orbital peak of density of states on the Zn-Al surface is the smallest (0.94), indicating that the interaction between O atoms and Zn-Al surface should be the weakest. This is consistent with the previous results of adsorption energy calculations.

### 3.3 Influence of O_2_ Coverage

**FIGURE 6:**
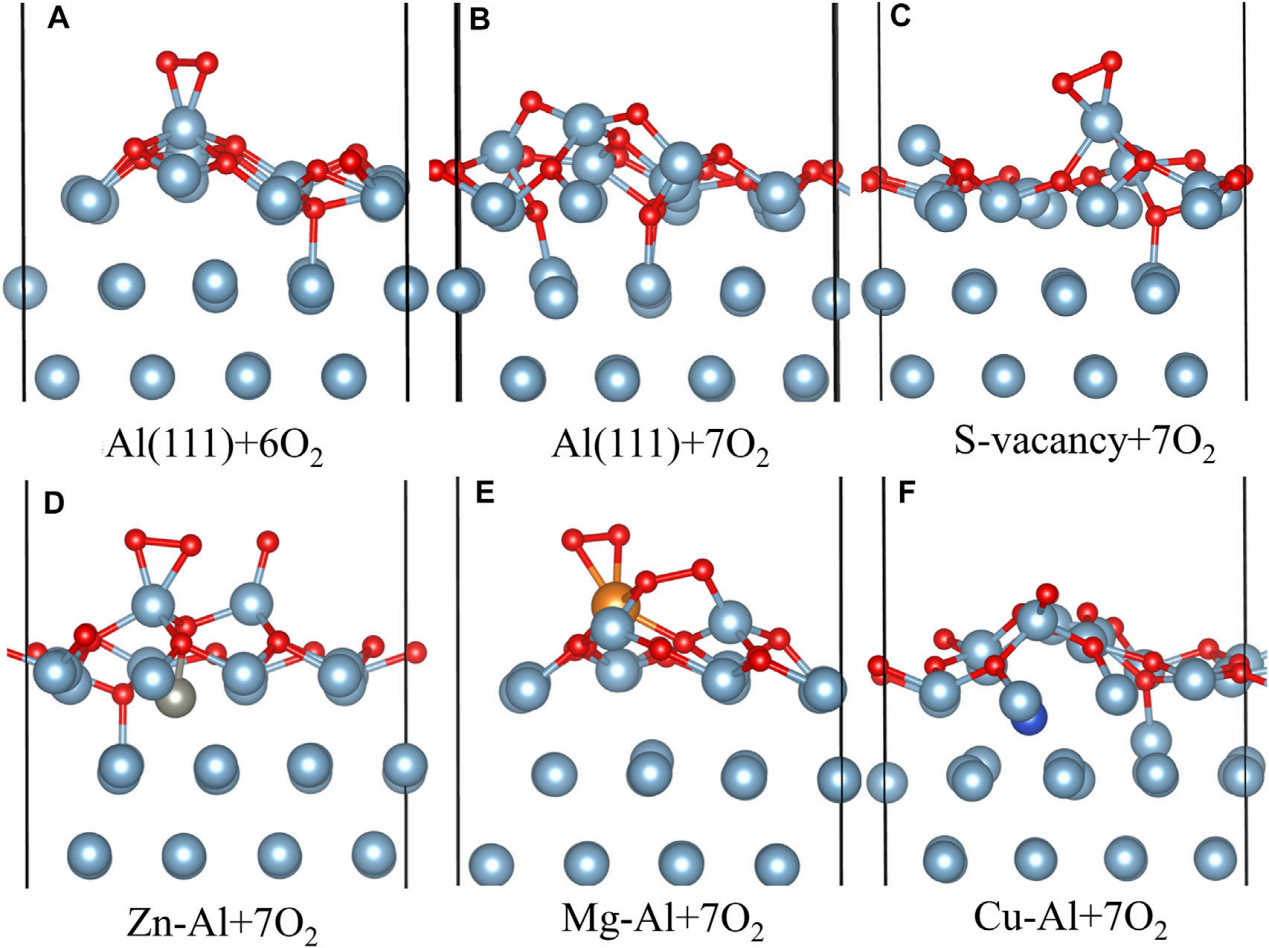
The side view of configuration of multiple O_2_ molecules adsorbed on each model surface.

It is well known that the oxidation reaction of aluminum surface is also affected by O_2_ coverage. O_2_ coverage refers to the ratio of the number of O atoms adsorbed on the surface to the number of surface atoms. In order to calculate the actual situation of O_2_ molecules adsorption on Al surface, in this paper, the adsorption behavior of O_2_ molecules under different coverages was also studied, and the adsorption energy, work function and charge transfer of O2 molecules on different model surfaces were calculated under the coverages from 1/8–1 ml. Table 3 lists the adsorption energies and charge transfer to O atoms for all the adsorption configurations under different coverages. It can be seen from the data in the table that the adsorption energy of the Al (111) surface drops suddenly when the sixth O_2_ molecule is adsorbed. This should be due to the fact that there are no fcc sites and hcp sites for adsorption on Al (111) surface at this time. And it is observed that O_2_ molecules are not dissociated on the surface at this time, as shown in Figures 6A,B. However, with the increase of O_2_ coverage, O_2_ molecules are dissociated and adsorbed on the Al (111) surface again, and the adsorption energy increases. In the actual surface oxidation reaction, because the oxidation reaction is very fast, it cannot be observed that O_2_ is not dissociated and adsorbed on the Al surface. If the non-dissociated adsorption of O_2_ molecule is ignored, the adsorption energy of O_2_ adsorption on Al (111) surface increases with the increase of O_2_ coverage, indicating that the adsorption capacity of the surface is enhanced with the increase of O_2_ concentration. However, the adsorption energy does not increase all the time. According to the calculation results of Baran et al. (Baran et al., 2014), the adsorption energy of O_2_ decreases slowly as the oxide film thickness on Al surface increases, until the adsorption energy is close to zero. At this time, at room temperature and normal pressure, O_2_ is not adsorbed, and the oxide film on Al surface reaches the limit thickness (about 9 layers thick, equivalent to ∼18 Å).

**TABLE 3:** Adsorption energies and charge transfer of O_2_ molecular adsorbed on surface of models.

O2 Coverage (ml)	Adsorption energy (eV)					Charge Transfer →O				
/Model	Al (111)	Zn-Al	Mg-Al	Cu-Al	S-Vacancy	Al (111)	Zn-Al	Mg-Al	Cu-Al	S-Vacancy
1/8	−8.5247	−7.9417	−9.1000	−8.2892	−8.9133	1.7461	1.5702	1.6823	1.6522	1.7229
2/8	−8.6491	−8.298	−8.9599	−8.6190	−8.7621	1.7415	1.6516	1.7060	1.6969	1.6984
3/8	−8.5679	−7.554	−8.7731	−8.9016	−8.7354	1.7199	1.5763	1.7121	1.6980	1.6993
4/8	−8.5773	−8.051	−8.7628	−8.6858	−8.7095	1.7137	1.6112	1.7048	1.7235	1.6963
5/8	−8.7949	−8.222	−8.9224	−8.1042	−8.8560	1.7056	1.7966	1.7069	1.6406	1.7114
6/8	−7.5950	−8.3855	−7.5972	−8.7542	−8.9681	1.5048	1.6519	1.4823	1.6790	1.6805
7/8	−8.8644	−7.5206	−7.0556	−8.7490	−7.9536	1.6683	1.6238	1.3639	1.6615	1.5170
1	−8.2869	−7.6357	−6.6684	−7.8144	−8.1762	1.5538	1.4573	1.3242	1.4988	1.5752

When there are doping atoms on the surface, the change of adsorption energy is more complicated. The adsorption energy is not only related to O_2_ coverage, but also to doping atom and the distance from surface doping atom. For example, the doping of Mg and Cu atoms and surface vacancy defects make adsorption energy near surface doping atoms and vacancies larger. The adsorption energy decreases with increasing distance from Mg, Cu atoms or vacancies, but increases again with increasing O_2_ coverage. It is worth noting that the adsorption energies of the Mg-Al and Zn-Al models drop abruptly and gradually decrease, when O_2_ coverage is greater than 5/8 ml. Observing the adsorption configuration of Mg-Al and Zn-Al, it is found that O_2_ molecules are not dissociated on the surfaces of Mg-Al and Zn-Al, after the fcc and hcp sites of surface are occupied by O atoms. This indicates that Mg atom promotes the dissociation adsorption of O_2_ on Al (111) surface under low O_2_ coverage (≤5/8 ml), while Mg atom inhibits the dissociation adsorption of nearby O_2_ molecules when O_2_ coverage is greater than 5/8 ml. The change of adsorption energy on Cu-Al surface is basically consistent with that on Al (111) surface, and the adsorption energy increases with the increase of O_2_ coverage. Observing the adsorption configuration of Cu-Al, it is found that Cu atoms are gradually embedded into the surface with the increase of O_2_ coverage. The structure of such a Cu-Al surface is similar to that of a surface vacancy defect, which can promote the adsorption of O_2_ molecules.

The strength of O_2_ adsorption is directly related to the amount of charge that is transferred to the O_2_ molecule. According to the charge transfer data in Table 3, when the O atom gets about 1.7 charge, the O_2_ molecule will be dissociated and adsorbed on the surface. And when the number of charges obtained by the atom is less than 1.6, the O_2_ molecules are physically adsorbed on the surface. The general trend is that the amount of charge transferred to O_2_ decreases as the O_2_ coverage increases. This is different from the calculated results of adsorption energy, but consistent with the research results of Baran et al. (Baran et al., 2014). This should be attributed to the fact that the adsorption energy is a consorted measure of interactions of the adsorbate with the surface, the image charge between the charged adsorbate and the metal, and changes in the interactions at the metal-oxide interface. When there are doping atoms on the surface, the amount of charge transferred to O_2_ changes. When Zn atom is doped, the amount of transferred charge decreases the most. When Mg atom is doped, and the O_2_ coverage is ≥6/8 ML, the amount of transferred charges also decreases abruptly. It shows that in these two cases, it is not conducive to the adsorption of O_2_.

In the calculation of Bader charge, it is also found that with the increase of O_2_ coverage, the charge obtained by the O_2_ molecule is not only from surface Al atoms, but also from underlying Al atoms. This charge transfer is called electron tunnelling from the underlying metal. And the possibility of transferring electrons from metal has been confirmed by experimental and theoretical calculations (Pacchioni et al., 2005; Giordano et al., 2008; Hellman and Grönbeck, 2008; Pacchioni, 2012). Pacchioni et al. (Pacchioni et al., 2005) believe that the metal-induced gap states (MIGS) between metals and oxides are the basis for electron transfer through the insulating layer, which promotes the adsorption behavior of ultrathin oxides.

**FIGURE 7:**
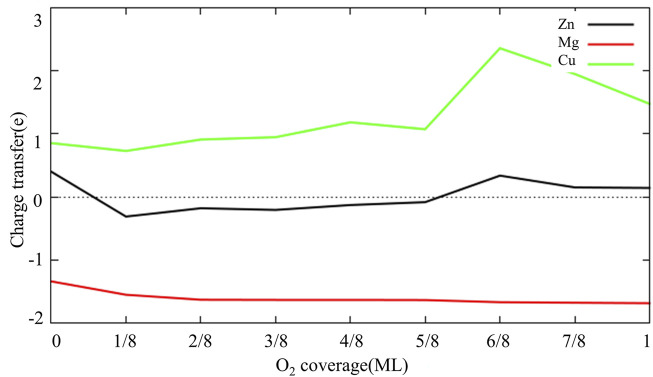
The charge transfer of Zn, Mg and Cu atoms as a function of O_2_ coverage (The electrons was lost below the dotted line, and electrons were gained above the dotted line).

Figure 6 shows the adsorption configuration of multiple O_2_ molecules on each model surface. As can be seen from Figures 6A,B with the increase of O_2_ coverage, O atoms adsorbed on the hcp site of Al (111) surface are gradually embedded into surface and bond with Al atoms in the subsurface layer. This is also consistent with the research results of Wei et al. (Wei et al., 2016). They believe that the oxide film of defect-free Al (111) surface is thickened by the migration of O atoms to the interior. It can also be seen from Figure 6 that Zn, Mg, Cu and surface vacancies delay the migration of O atoms to the subsurface. In particular, Mg atoms inhibit the migration of surrounding O atoms to the subsurface. This may be related to the fact that Mg atoms are gradually pulled out of the surface with increasing O_2_ coverage. Figure 7 shows the charge changes of Zn, Mg and Cu atoms with O_2_ coverage. Comparing Figures 6, 7 it can be seen that with the increase of O_2_ coverage, Mg atoms lose more electrons and are pulled out of the surface, while Cu atoms gain more electrons and migrate to the interior. Zn atoms change between losing and gaining electrons, so Zn atoms move near the surface. In addition, O_2_ adsorption results in pronounced structural reconstructions. The Al atom directly below the O_2_ adsorption site is dragged out from the surface. Baran et al. (Baran et al., 2014) believe that this rearrangement results in the formation of Al vacancies in the aluminum slab. Such slab structure of Al vacancies should be correspond to the tetrahedral coordination of Al in the surface oxides. Thus, it can be seen from the above results that the difference in electronic properties of the doping atoms on surface leads to the difference in structure and thickness of oxide film on surface of aluminum alloy.

The work function of each model adsorbing multiple O_2_ molecules is also calculated. It can be seen from Table 4 that the adsorption of O_2_ molecules on Al (111) surface has a great influence on the work function, and the work function increases with the increase of O_2_ coverage. This indicates that with the adsorption of O_2_ molecules, the electron activity on surface of aluminum alloy is gradually weakened, and the more difficult it is for electrons to escape from surface, the higher the work function. However, surface doping of Zn, Mg and Cu atoms has an effect on the adsorption behavior of O_2_, resulting in the work function being different from that of Al (111). Among them, Zn and Cu atoms have a greater influence on the work function, while Mg and vacancy defects have less influence on the work function. With the increase of O_2_ coverage, Zn atoms make the work function increase faster. When the O_2_ coverage is less than or equal to 5/8 ml, Cu atom causes the work function to increase rapidly. But, under the high coverage of O_2_, Cu atoms are gradually embedded into surface, resulting in a lower work function than that of Al (111) surface. The influence of doping atoms on the work function should be related to the difference of the work function between doping atoms and the matrix. The smaller the difference is, the greater the influence on the work function is. The difference of surface work function can have a huge impact on subsequent hydration and corrosion reactions of surface.

**TABLE 4:** Work function of O_2_ molecular adsorbed on surface of models.

O_2_ coverage (ML)	Work function (eV)				
/Model	Al (111)	Zn-Al	Mg-Al	Cu-Al	S-Vacancy
0	4.0406	4.0445	4.0407	4.0542	4.0553
1/8	4.0519	4.1817	4.0606	4.1548	4.0714
2/8	4.0748	4.2035	4.0854	4.2111	4.2157
3/8	4.1248	5.7497	4.0372	4.2856	4.2732
4/8	4.1546	5.7711	4.0988	4.2635	4.3308
5/8	4.0723	5.8348	4.0896	5.8056	4.4145
6/8	5.3607	5.7399	5.3034	4.0956	4.2935
7/8	4.7768	6.3315	5.4161	4.2711	5.4185
1	5.1476	5.3868	5.4332	4.8565	4.6764

## 4 Conclusion

In this paper, the doping of alloying elements (Zn, Mg, Cu) on Al (111) surface and the adsorption of O_2_ molecules on doping surface were calculated by using the first-principles method based on DFT. The formation process of oxide film on doping Al (111) surface was studied. The following conclusions are drawn.

(1) Zn, Mg and Cu atoms can stably combine with Al atoms on Al (111) surface, and the stability depends on types of doping atoms and their electronic properties. Due to the difference in the atomic radius and number of electrons of Zn, Mg, and Cu, the surface flatness is different.
(2) O_2_ molecules can spontaneously dissociate and adsorb on the fcc and hcp sites of the Al (111) surface, and the degree of dissociation and adsorption is related to surface doping atoms and O_2_ coverage. Under low O_2_ coverage (≤5/8 ml), Mg and vacancy defects can promote the adsorption of O_2_, and Zn and Cu atoms delay O_2_ adsorption. Under high O_2_ coverage (>5/8 ml), Zn and Mg atoms delay O_2_ adsorption. Meanwhile, the increase of O_2_ coverage promotes its adsorption behaviour due to the electron tunnelling of underlying metal.
(3) With the increase of O_2_ coverage, O atoms adsorbed on the hcp site of Al (111) surface gradually migrate to subsurface layer and bond with the subsurface Al atoms. Doping atoms Zn, Mg, Cu and vacancy defects hinder the migration of surrounding O atoms to the subsurface, resulting in different structures and thicknesses of surface oxide film surround doping atoms.
(4) Compared with Al atoms, the ability of Zn, Mg, and Cu atoms to gain or lose electrons determines their positions on surface. The ability of Cu atom to obtain electrons is relatively strong, resulting in Cu atom embedded into surface. The ability of Mg atom to lose electrons is relatively strong, resulting in Mg atom to be pulled out of surface. The ability of Zn atom to gain or lose electrons is similar to that of Al atom, resulting in Zn atom to sit near the surface.
(5) The surface work function is affected by O_2_ coverage and doping atoms. The surface work function of Al (111) increases rapidly when Zn and Cu atoms are doped on surface.

It can be seen from the above research results that Zn and Mg are not conducive to the oxidation reaction of Al surface, while Cu and vacancy defects can promote the oxidation on Al surface. The doping of alloying elements results in different surface oxide structures. The surface structure can be controlled by adding alloying elements to the Al base, thereby achieving the purpose of changing the surface activity and adjusting the thickness of oxide film. Therefore, the research results can provide theoretical reference for the design of shell material for lithium-ion battery.

## Data Availability

The original contributions presented in the study are included in the article/supplementary material, further inquiries can be directed to the corresponding author.
